# Synthesis and application of superabsorbent polymer microspheres for rapid concentration and quantification of microbial pathogens in ambient water

**DOI:** 10.1016/j.seppur.2020.116540

**Published:** 2020-05-15

**Authors:** Xunyi Wu, Xiao Huang, Yanzhe Zhu, Jing Li, Michael R. Hoffmann

**Affiliations:** Linde+ Robinson Laboratories, California Institute of Technology, Pasadena, CA 91125, United States

**Keywords:** Super-absorbent polymer (SAP), Concentration method, Waterborne pathogen, 3D printing, Point-of-sample collection

## Abstract

Even though numerous methods have been developed for the detection and quantification of waterborne pathogens, the application of these methods is often hindered by the very low pathogen concentrations in natural waters. Therefore, rapid and efficient sample concentration methods are urgently needed. Here we present a novel method to pre-concentrate microbial pathogens in water using a portable 3D-printed system with super-absorbent polymer (SAP) microspheres, which can effectively reduce the actual volume of water in a collected sample. The SAP microspheres absorb water while excluding bacteria and viruses by size exclusion and charge repulsion. To improve the water absorption capacity of SAP in varying ionic strength waters (0–100 mM), we optimized the formulation of SAP to 180 g⋅L^−1^ Acrylamide, 75 g⋅L^−1^ Itaconic Acid and 4.0 g⋅L^−1^ Bis-Acrylamide for the highest ionic strength water as a function of the extent of cross-linking and the concentration of counter ions. Fluorescence microscopy and double-layer agar plating respectively showed that the 3D-printed system with optimally-designed SAP microspheres could rapidly achieve a 10-fold increase in the concentration of *Escherichia coli* (*E. coli*) and bacteriophage MS2 within 20 min with concentration efficiencies of 87% and 96%, respectively. Fold changes between concentrated and original samples from qPCR and RT-qPCR results were found to be respectively 11.34–22.27 for *E. coli* with original concentrations from 10^4^ to 10^6^ cell·mL^−1^, and 8.20–13.81 for MS2 with original concentrations from 10^4^ to 10^6^ PFU·mL^−1^. Furthermore, SAP microspheres can be reused for 20 times without performance loss, significantly decreasing the cost of our concentration system.

## Highlights

• A portable, hand-pressed 3D-printed system with SAP microspheres was developed.
• This system could achieve efficient concentration of environmental microorganisms.
• Superior performance was achieved with varying ionic strengths in a short time.
• Optimized SAP microspheres could be reused 20 times with simple procedures.

## Graphical abstract

**Figure:**
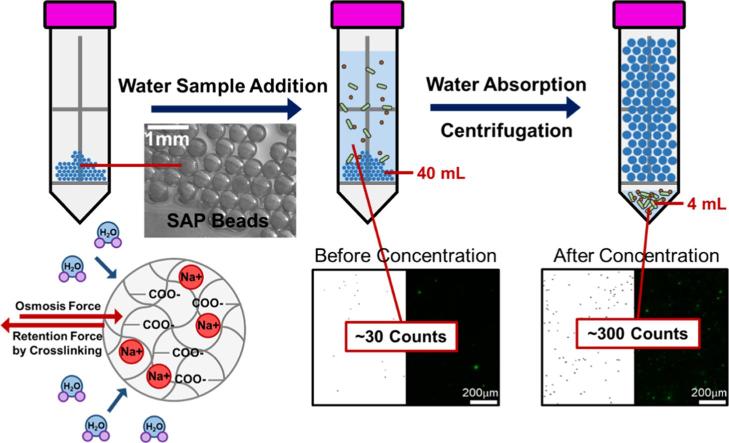

## 1 Introduction

Waterborne pathogens, including various pathogenic bacteria, viruses, and protozoa, are responsible for a series of diseases, and thus have been a major public health concern worldwide [1], [2], [3]. According to the World Health Organization (WHO), global mortality attributable to water-related diseases is currently 3.4 million per year, most of which are children [4]. This issue is especially severe in developing regions of the world due to the scarcity of clean water supplies and poor sanitation conditions [1], [4], [5], [6]. Sensitive detection and quantification methods for waterborne pathogens, including traditional culture-based methods, or more recently, nucleic acid amplification tests [3], [7], [8], [9], [10], are thus indispensable to ensure water safety and to protect the public health.

Testing for pathogens in environmental waters has two main challenges: (1) the concentrations of pathogens in environmental water samples are usually magnitudes lower than those in clinical samples; and (2) the small sample volume being analyzed in each assay makes the direct detection of pathogens in environmental water samples nearly impossible [1], [3]. Pathogen concentrations below the detection limit of the methods mentioned above, do not guarantee the safety of water, as they may still pose a health risk considering their low infectious doses [5], [11].

Numerous techniques for pathogen concentration have been developed. Traditional techniques including polyethylene glycol (PEG) coagulation and precipitation, membrane filtration, centrifugation, and evaporation are most commonly used [12], [13]. However, these concentration methods require complicated setups and are often time-consuming, which means water samples have to be transported to centralized laboratories with inevitable sample degradation even under continuous cold chain [1]. For field-studies, marine biologists use three steps of Tangential Flow Filtration (TFF) to concentrate water samples with a volume of 120 L [14]. The use of filtration cartridges and membranes, as well as pumping systems, are inevitable and the first TFF step for 60-fold concentration alone takes four hours [15]. The Bag-Mediated Filtration System (BMFS) provides another in-field concentration method that uses gravity as the driving force to filter and concentrate water samples. However, filters and an elution step followed by PEG/NaCl precipitation were also required [16]. Some new techniques are emerging, such as in-plane evaporation [17], magnetic nanoparticle platform on chip [18] or magnetic separators [19], [20]. However, these new methods are still limited to laboratory use and are incapable of handling field samples with volumes of at least 1 or 2 L [19], [20], [21].

Super-absorbent polymer (SAP) microspheres are a class of cross-linked hydrogels that can absorb and retain water up to 1000 times the initial dry weight of the SAP beads [22], [23]. SAP materials are widely used in personal disposable hygiene products (e.g., diapers), and for agricultural water preservation or waste fluid spill control [24], [25]. By controlling the pore sizes of the hydrogel down to several nanometers, SAPs can absorb water but at the same time exclude particles with sizes above several nanometers, such as bacteria and viruses [24], [26]. In order to use SAPs for microbial sample concentration, the SAPs were synthesized as small spherical microspheres using a milli-fluidic flow system. Itaconic acid is added to the polymer to obtain negatively charged polymeric microspheres that have uniform spherical shapes, which minimize electrostatic adsorption of microorganisms on the surface of the microspheres [27].

SAP microspheres absorb water through osmosis, which is driven by polyelectrolyte counter ions attached to the polymer. However, the extent of water absorption is limited by the retention force of the polymer networks due to cross-linking. The maximum water absorbencies and water absorption rates of the SAPs are determined by the equilibrium of the osmotic forces and the retention forces. For a given SAP formulation with a fixed number of polyelectrolyte counter ions, the osmotic force generated by the SAPs decreases with an increase of ionic strength, which effectively lowers the maximum water absorbency and water absorption rate of a specific SAP formulation. Therefore, the ionic strength of environmental water samples may have a significant impact on the performance of the SAP microspheres.

Here we have adjusted the composition of the SAP microspheres to achieve optimal performances in freshwater or saline waters and further demonstrated that bacteria and viruses collected from environmental water samples can be rapidly concentrated using optimized SAP microspheres. We have further developed a 3D-printed portable, hand-pressed centrifuge system to realize the single-step concentration using SAP microspheres for onsite water concentration in limited-resource settings and without trained personnel. Our study highlights that concentration of the microbial samples using SAPs provides an alternative sample concentration method that avoids a typical multi-step procedure that is often tedious, time-consuming, and inappropriate for use in underdeveloped parts of the world.

## 2 Materials and methods

### 2.1 SAP preparation and characterization

Monomers used for synthesis of the polymeric beads were acrylamide and itaconic acid, which were dissolved in deionized water with concentrations of 180 g⋅L^−1^ and 20 g⋅L^−1^, respectively. Bis-acrylamide (4.0 g⋅L^−1^) was added to the monomer solution as a cross-linker and potassium persulfate (2.6 g⋅L^−1^) was added as the initiator of the polymerization reaction [27], [28], [29]. Itaconic acid in the monomer solution was fully neutralized by sodium hydroxide prior to the polymerization. All chemicals were purchased from Sigma-Aldrich and were used as received.

**Fig. 1:**
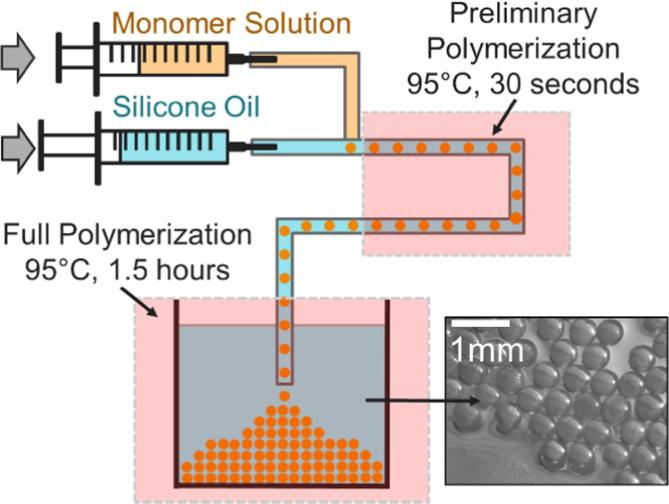
A schematic illustration of the synthesis steps producing SAP microspheres.

SAP microspheres with diameter of 500 µm were prepared by a two-step polymerization using a milli-fluidic system as shown in Fig. 1. Droplets of the monomer solution were generated through a T-junction with an inner diameter of 1/16 in. into the carrying silicon oil of 500 cSt. For the generation of water phase droplets, oil phase and water phase were injected at 0.5 mL⋅min^−1^ and 0.2 mL⋅min^−1^, respectively, using two syringe pumps (74905-02, Cole-Parmer, US), into the tubing with 1/16-inch inner diameter. Generated droplets first underwent preliminary polymerization in the tube for 30 s at 95 °C. Subsequently, full polymerization of the microspheres was achieved after the microspheres left the tube and settled in the hot oil bath at 95 **°**C for 1.5 h. This system can generate microspheres of diameters ranging from 500 µm to 2000 µm. Another fabrication method, inverse suspension polymerization, can be used to generate microspheres of diameters ranging from 10 µm to 500 µm, which can be used in smaller concentration systems with smaller starting sample volumes (see Fig. S1). After the polymerization, fabricated microspheres were washed using 95% ethanol to wash off residual oil. Microspheres were soaked in DI water for 24 h to remove any remaining monomers and subsequently dried under vacuum overnight. Weight analyses of dried SAP microspheres were performed using an analytical balance (AT469, Mettler, USA).

### 2.2 Water absorbency evaluation

The water absorbency Q (g/g) is defined as the swollen weight of SAP (g) divided by the dried weight of SAP (g). To simplify the experimental procedures and to evaluate the water absorbency more easily and precisely, larger SAP blocks (~1 × 10^−2^ g/block) (Fig. S7) were fabricated with varying monomer and cross-linker ratios (see Table 1). SAP blocks were fabricated under the same condition for SAP beads fabrication, and they share the same adsorption properties with SAP beads. Na^+^ content in the polymer was changed by varying the proportion of sodium itaconate in the monomer solution. SAP blocks were tested for their absorbency in sodium chloride solutions with a series of ionic strengths of 0, 100, 200 and 500 mmol⋅L^−1^ [30]. The ionic strength *S* of all solutions was calculated using the following equation:

(1) $$S=\frac{1}{2}\sum_{i=1}^{n}{c_{i}z_{i}}^{2}$$

where *c* is the concentration of the dissolved salt ion in mol⋅L^−1^, and *z* is the valence of the ion. For the dissolved salts, a complete dissociation was assumed [30]. After absorbing water overnight, polymer blocks were drained and the remaining water on the surface of the SAP was gently removed with a paper tissue. The weight of the fully swollen SAP blocks was determined, and their corresponding water absorbency (gram water absorbed by gram dried polymer) was calculated.

**Table 1:** SAP recipes with varying cross-linking degree and sodium content.

	Acrylamide (g⋅L^−1^)	Itaconic Acid (g⋅L^−1^)	Bis-Acrylamide (g⋅L^−1^)
(O1) Original Recipe	180	20	4
C1	180	20	0.2
C2	180	20	0.4
C3	180	20	1
C4	180	20	2
S1	180	50	4
S2	180	75	4
S3	180	100	4

To measure the absorption rate, completely dried SAP microspheres were soaked in water. Their diameter changes upon swelling were recorded and measured with a light microscope (Leica M205FA, Leica Co., Germany). The water absorption rates were evaluated by three models with MATLAB (see supplementary information) and compared to the experimental results.

### 2.3 Microbial sample preparation

*E. coli* (ATCC 10798) was used as model bacteria in this study and cultured in Luria-Bertani broth (BD Difco™, USA). Before each concentration test, cells were harvested, washed and serially diluted to 10^4^–10^6^ cells⋅mL^−1^ using phosphate-buffered saline (pH 7.4) (Corning™, USA). Coliphage MS2 (ATCC 15597-B1) was chosen as model virus. The growth and purification procedures of MS2 are described in our previous work [10]. Before spiking MS2 in water samples, host *E. coli* cells were removed through centrifugation at 12000 rpm (13523 g) for 2 min (Eppendorf 5424, US). Briefly, MS2 suspension was diluted to 10^5^–10^7^ PFU⋅mL^−1^ for seeding studies. Environmental water samples were collected from a turtle pond on the Caltech campus and from the primary effluent from a local wastewater treatment plant (with ionic strengths of 15 and 20 mmol⋅L^−1^, respectively [31]). The conductivities and pH values of environmental water samples were measured with an electrical pH/conductivity meter (Orion Star A215, Thermo Scientific, US) and ionic strengths were quantified using Griffin’s equation [32].

### 2.4 Concentration experiments

A manual hand-powered tube system was designed and fabricated for field use in resource-limited settings (see Fig. 4). A 3D-printed filter with a mesh size of 300 µm (Fig. S4A) was inserted into a 50 mL commercial centrifuge tube (SuperClear™ Ultra High Performance Centrifuge Tubes, VWR, USA). The filter was fabricated using a high-resolution 3D printer (ProJet™ MJP 2500 Plus) with Visijet M2 RCL Clear Material (3D Systems, Rock Hill, SC). Subsequently, the tube was divided into two chambers: the upper chamber (filled with 0.5 g SAP microspheres) for sample concentration; and the lower chamber for concentrated sample collection. 40 mL water sample was added into the tube and was kept in the upper chamber. The sample water would not enter the lower chamber through the filter due to the surface tension of the liquid. The tube was left standing for 15 min for SAP microspheres to absorb water. Then the residual water (~4 mL) was transferred to the lower chamber by centrifugation (~500 rpm). The hand-press centrifuge was adapted from a commercially-available salad spinner (32480, OXO, USA). The filter and microspheres were taken out of the centrifuge tube. Subsequently, the concentrated sample was collected and its volume was measured. The concentrations of *E. coli* and MS2 in samples before and after concentration were measured and compared as described in Section 2.5. Concentration experiments of *E. coli* solutions with initial concentrations of 10^4^, 10^5^ and 10^6^ cell·mL^−1^ were performed as independent triplicates. The difference before and after each microsphere-concentration experiment was compared using qPCR assays. The qPCR assays of *E. coli* solutions of 10^5^, 10^6^ and 10^7^ cell·mL^−1^ were also performed as positive controls. Concentration experiments using MS2 with initial concentrations of 10^5^, 10^6^ and 10^7^ PFU·mL^−1^ were performed in triplicate. The RT-qPCR assays of MS2 solutions of 10^6^, 10^7^ and 10^8^ PFU·mL^−1^ were also performed as positive controls.

### 2.5 Concentration efficiency analyses

In this study, we use concentration efficiency to evaluate the performance of the concentration system. Here, we define the concentration efficiency as the percentage of microorganisms that remain in concentrated samples. Concentration efficiencies for *E. coli* and MS2 were analyzed using both of microcopy and culturing methods at the level of cell. The performance of the system was further evaluated by the fold-change using PCR-based molecular methods. *E. coli* cell concentrations were quantified using fluorescence microscopy (Leica DMi8, Leica Co., Germany) after SYBR-Green (Invitrogen™, USA) staining according to the manufacturer’s protocol [10]. Fluorescence pictures were processed and the cell numbers were counted by ImageJ software (ImageJ 1.51j8, Wayne Rasband National Institutes of Health, USA). The number of *E. coli* was also evaluated by plating on Luria-Bertani agar (BD Difco™, USA). Colonies were counted after 14 h of incubation at 37 °C. Total environmental bacterial concentrations in environmental water samples (pond water and wastewater) were enumerated by fluorescence microscope counting and plate counting on LBA as well. The MS2 concentration was determined by the double agar layer method [33].

Concentration efficiencies of *E. coli* and MS2 were quantified by quantitative PCR (qPCR) and quantitative reverse transcription PCR (RT-qPCR) using a 6300 Realplex4 qPCR platform (Eppendorf, Hamburg, Germany). Relevant primer sets and probes are listed in Table S1. For *E. coli*, the qPCR assay targeting the 16 s rRNA gene was carried out in a 20-μL reaction mixture consists of 10 μL PerfeCTa® qPCR ToughMix® (Quanta BioSciences Inc.), 0.25 μM forward primer, 0.25 μM reverse primer, 0.25 μM TaqMan probe, 2 μL of template DNA, and nuclease-free-water. The qPCR thermocycling involves 3 min of initialization at 95 °C, and 40 cycles of denaturation at 95 °C for 15 s followed by annealing/extension at 55 °C for 30 s. For MS2, the RT‐qPCR reactions were performed using QIAGEN OneStep RT‐PCR Kit (Germantown, MD). Each 25-µL reaction mix included 800 nM forward and reverse primers, 300 nM TaqMan probe, 0.5 mg·mL^−1^ BSA, 1x RT‐PCR buffer, 0.4 mM dNTP, 1 U enzyme mix, 3 µL of template RNA, and nuclease-free water.[10] The RT‐qPCR thermocycling involves an initial reverse transcription step at 50 °C for 30 min, followed by an initial denaturation at 95 °C for 15 min, then 45 cycles of 94 °C for 15 s and 60 °C for 60 s. The nuclease-free water was used as negative controls for all qPCR and RT-qPCR assays. Here for each concentration assay, the concentration efficiency was evaluated by the fold change value:

(2) $$\mathrm{Fold}\;change=\frac{C(aftertheconcentration)}{C(beforetheconcentration)}\times 100\%$$

where *C_(before the concentration)_* and *C_(after the concentration)_* are concentrations of sample before and after concentration calculated with standard curves performed on each plate. Concentrations of *E. coli* and MS2 standard samples were respectively evaluated using the fluorescence microscopy and the double-layer agar as described in Section 2.5. All qPCR and RT-qPCR reactions performed in this study reached efficiency between 90% and 110%, indicating the high reliability of our performed assays [34]. Quantification data of samples before and after concentration experiments for the fold change calculations for both *E. coli* and MS2 can be found in Table S3 in the supporting information. All samples were run in triplicate.

### 2.6 Reusability test

To reuse the SAP microspheres after the concentration tests, the microspheres were washed under running tap water for two minutes to remove the remaining bacteria and viruses from the surfaces of the microspheres. The SAP microspheres were subsequently washed in 30 mL Milli-Q water and followed by being dried for subsequent reuse. The synthesized SAP microspheres were fully loaded with water via absorption and then dried using a vacuum oven (VO914A, Thermo Scientific, USA) for 20 consecutive cycles. The gross weights and water absorbencies were measured to test their reusability after successive swelling and drying cycles.

## 3 Results and discussion

### 3.1 Synthesis of SAP microspheres

Uniform poly (acrylamide-co-itaconic acid) (P(AM-co-IA)) microspheres were fabricated using a system as illustrated in Fig. 1. Monomer solution-in-oil droplets were generated with two syringe pumps, using a T-junction. After the generation of monomer solution droplets, the P(AM-co-IA) microspheres required at least 1.5 h at 95 °C to achieve complete polymerization: the polymerization reaction was catalyzed by free radicals from persulfate generated by heating and dissociating potassium persulfate. The persulfate free radicals convert monomers of acrylamide and itaconic acid with double bonds to free radicals that react with other monomers to begin the polymerization chain reaction. The elongating polymer chains are randomly cross-linked by bis-acrylamide, resulting in a gel matrix structure [35]. The two-step polymerization system was designed such that the polymer microspheres would only undergo preliminary polymerization in the tube, so they would not fuse into each other and block the tube. When the partially polymerized microspheres left the tube, they were immersed in an oil bath for 1.5 h allowing for complete polymerization. The characteristics of washed and fully-dried SAP microspheres presented uniform spherical shape with a characteristic diameter of 500 ± 8 µm, white color, and smooth surfaces as shown in Fig. 1. Each SAP microspheres have the same formula and are formed with the same amount of monomers, being very uniform after absorbing water. The slight difference in the shape of the sphere when they are dried was most likely due to the inconsistent shape change during the drying process. When the microspheres were fully dried, their density was slightly lower than that of water due to that voids presented in the polymer structure. Variances in the porous polymer structure during drying of each polymer microspheres may also lead to slight density inconsistency between microspheres, but these slight differences in shape and density would not influence the performance of SAP microspheres on water absorption as they became uniform after they start to absorb water. Smaller size microspheres can be fabricated by inverse suspension polymerization method and shared similar SAP properties (see Fig. S1B).

### 3.2 Optimization of SAP for various water matrices

**Fig. 2:**
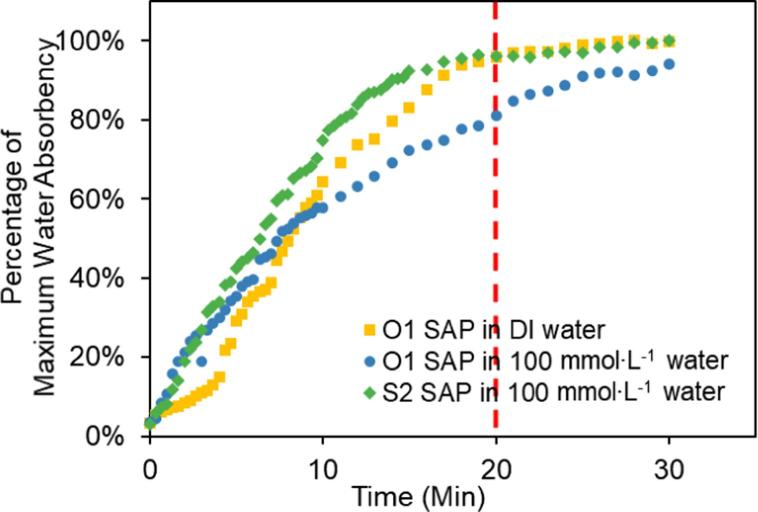
Water absorbency of original microspheres (O1) and revised microspheres (S2) in DI water and saline water (100 mmol⋅L^−1^) over time.

SAP microspheres used in the previous research with fixed composition can only work in deionized water, since both the maximum capacity, and the rate of water absorption would decrease drastically in high ionic strength water. Hence, the composition of the SAP beads needs to be adjusted to achieve optimal performances for different water matrices. SAP blocks fabricated according to the original monomer solution recipe (180 g⋅L^−1^ AM, 20 g⋅L^−1^ IA and 4.0 g⋅L^−1^ Bis-A) could absorb water of around 80 times their own weight (water absorbency (Q ~ 80), and a maximum absorbency of 96% was reached under 20 min in DI water (see Fig. 2). Although the polymer is stable and tolerant to different environmental conditions, the maximum water absorbency and water absorption rate of the polymer were significantly reduced in higher ionic strength water samples due to the decreased osmotic force. For environmental waters, the average ionic strength of freshwater and wastewater are around 5 mmol⋅L^−1^ and 50 mmol⋅L^−1^, respectively, and can be as high as 150 mmol⋅L^−1^ for untreated wastewater [36], [37], [38], [39]. In water with an ionic strength of 100 mmol⋅L^−1^, the same SAP’s absorbency decreased to 30% of its maximum absorbency. Less than 80% of maximum water absorbency was achieved, and equilibrium could not be reached for more than 30 min (see Fig. 2). Therefore, the SAP composition requires optimization to improve its performance in saline water.

The water absorbency of SAP is determined by the balance of three forces: (1) the osmosis potential between the solution within the polymer network and the external solution; (2) the electrostatic repulsion resulting from the fixed charges on the polymer chains; and (3) the elastic retractile response of the polymer network [40]. Forces (1) and (2) increase the absorption of SAP while force (3) restricts the absorption. The high sodium cation (polyelectrolyte counter ion) concentration within the polymer network provides osmotic pressure, which quickly drives water into the polymer. As the water penetrates the polymer, the sodium cation is diluted, and the concentration of sodium cation in the polymer decreases, leading to a decrease of osmotic force [22], [23]. At the same time, the retention force of the polymer is increasing with the expansion of the polymer network. When the balance between the osmotic force and retention force is reached, the SAP is at equilibrium. For the cross-linked polymer, the water absorbency, *Q*, can be expressed as a function using elasticity gel theory of Flory [35], [40], which has the following form:

(4) $$Q^{\frac{5}{3}}=\left[{\left(\frac{i}{2V_{u}S^{\frac{1}{2}}}\right)}^{2}+\left(\frac{1}{2}-X_{1}\right)V_{1}\right]V_{e}/V_{0}$$

where *Q*: maximum water absorbency (g/g); *V_e_/V_0_*: crosslinking density of polymer (amount cross-linker/total polymer); *(1/2 − X_1_)/V_1_*: affinity between polymer and external solution (*X_1_:* interaction parameter of polymer with solvent*; V_1_*: molar volume of solvent in a real network); *V_u_*: volume of structural unit; *i*: electronic/ionic charge present on the polymer backbone per polymer unit; *i/V_u_*: fixed charge per unit volume of polymer; *S*: Ionic strength of external solution (mol⋅L^−1^). Since the affinity of the polymer to water does not change in our case, and the volume of the structural unit is fixed, the maximum water absorbency is solely controlled by the crosslinking density, fixed-charge density and external ionic strength.

Two methods were explored to improve the performance of SAP in water at different ionic strengths: one was to reduce the retention force of the polymer by decreasing the cross-linking degree; and the other was to increase the osmotic pressure by increasing the sodium content in the polymer. The recipe changes of SAP also varied the pore size of the fabricated SAP, which was still small enough to exclude bacteria and viruses with high concentration efficiencies (see Section 3.4 for results and discussion).

**Fig. 3:**
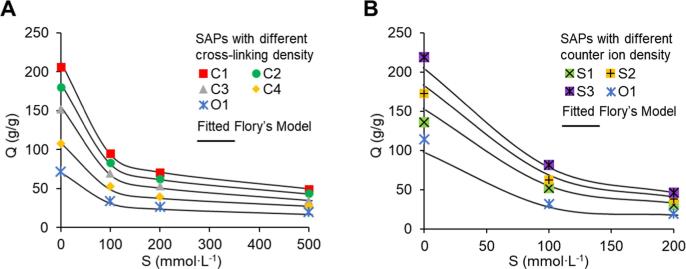
Change of maximum water absorbency (Q) vs. ambient ionic strengths (S), and the impacts of changing cross-linking density (A) and counter ion density (B) on maximum water absorbency. Error bars are all smaller than 1% and are not shown on graphs.

Fig. 3 shows the change of SAP absorption performance induced by varying cross-linking degrees and counter ion concentrations. As shown in Fig. 3A, SAP with the lowest cross-linking degree (C1) could reach water absorbency of 50 in the highest ionic strength solution (500 mmol⋅L^−1^), while the absorbency of the original microspheres (O1) decreased to less than 20. However, it should be noted that when loosening the structure of the polymer to reduce the retention force, the mechanical strength of the SAP is also reduced. If the cross-linking degree were modified to an amount smaller than 1 g Bis-A per 1000 g total monomer, then the SAP microspheres broke easily during the centrifugation step and the debris of the broken SAP microspheres entered the residual water sample, influencing the experimental results. Thus, broken SAP microspheres cannot be reused.

Increasing the Na^+^ content in the polymer also significantly improved the absorption rate of SAP in saline water, by providing an increased osmotic force (see Fig. 3B). Before the centrifugation step, the microspheres needed to reach at least 90% of their maximum absorbency. At this stage, the absorption rate slows down and the weight of SAP did not change a lot (Fig. 2), which was important for the following centrifugal step. For a successful concentration step, a small volume of sample must remain after the water absorption through SAP. Therefore, a slow water absorption rate of SAP microspheres during centrifugation would be desirable. Otherwise, the SAP microspheres would continue to rapidly absorb the remaining water during centrifugation and the sample water could be totally absorbed by SAP microspheres at a fast absorption rate, leading to the failure of the concentration process. For the original SAP microspheres, less than 80% of the maximum water absorbency was obtained at 20 min in 100 mmol⋅L^−1^ water while still swelling rapidly. If we were to use SAP microspheres made with this recipe, the concentration process would take more than 30 min. However, the microspheres with the S2 recipe would reach 95% maximum water absorbency in 20 min, which was much faster than the microspheres with the original recipe (~35 min). The improvement of the absorption rate was further confirmed using three models (see supplementary information). By applying the models to our experimental data to calculate the diffusion coefficients, all three models show the increase of the diffusion coefficients by around 50% after using the optimized recipe. Since the resulting linear fits of *Q*^5/3^ versus the cross-linking density and the fixed charge density (*i/V_u_*) are consistent with the predictions of the Flory theory [39], [40] (Fig. 3), the SAP formulations could be easily customized to suit different ionic strengths of the respective water matrices.

### 3.3 Tube concentration system

**Fig. 4:**
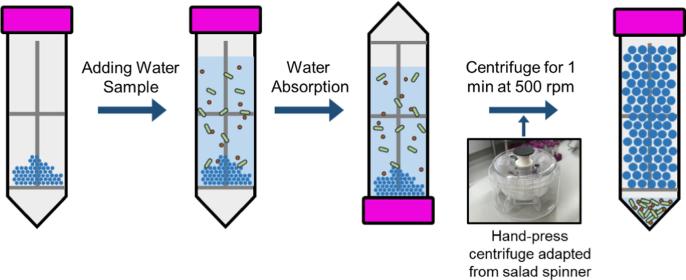
The tube system designed for microbial pathogen concentration using SAP microspheres. The tube is composed of 0.5 g SAP microspheres and a 3D-printed filter. After adding the water sample, the tube is left to stand for 20 min for the full absorption of water by SAP. Non-absorbed water is pushed to the lower chamber using a hand-press centrifuge.

Furthermore, the previous concentration method introduced in Xie et al. (2015) required five manual and consecutive operations of using pipettes to collect concentrated samples (each step concentrating about 20% of the sample volume), which made this approach tedious, time-consuming and not applicable in field. Therefore, our study remarkably developed a portable, hand-pressed centrifuge system with one-step operation to facilitate the efficient use of SAP beads for onsite concentration for waterborne microorganism in low-resource settings, thus allowing our concentration method to be easily performed by people without any prior training. Fig. 4 schematically illustrates the tube system for microbial pathogen concentration. Each tube contains 0.5 g SAP microspheres and a 3D-printed filter. The 3D-printed filter divided the tube into two chambers and the water samples are restricted in the upper chamber before centrifugation by the filter due to the surface tension of the sample. After adding the sample, the tube only need to be left to stand for 20 min for the full absorption of water by the SAP. Non-absorbed water is transferred to the lower chamber using a hand-press centrifuge. After 20 min, more than 90% of the sample was adsorbed and continued absorption became very slow. Thus, a remaining water sample (~4 mL) could be collected by centrifugation. The hand-press centrifuge was adapted from a salad spinner, which can reach an average rotation speed of 500 rpm. This spinning speed was fast enough, as evident, as the concentration efficiency (percentage of microorganisms recovered after concentration) did not change when using a commercial centrifuge with up to 1200 rpm (data not shown). This hand-pressed spinner reduced the cost of the system and made the system totally off-grid and suitable for field use. Moreover, our system may be a promising tool in field studies, as it can rapidly concentrate environmental samples. One example of applications could be in-field sequencing when coupled with the new sequencing technology, MinION sequencer [41].

### 3.4 Microorganism concentration performance

**Fig. 5:**
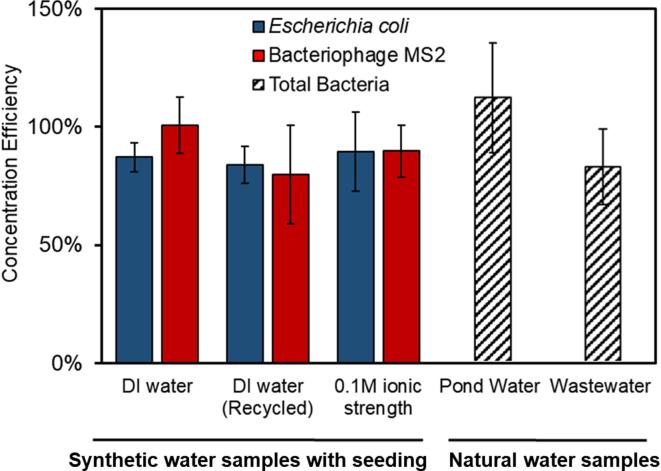
Concentration efficiencies of *E. coli*, MS2 and total bacteria using the tube concentration system calculated by microscopic cell counts, plague forming unit quantification. *E. coli* and MS2 were concentrated using new SAP microspheres and recycled SAP microspheres after 20 drying- swelling cycle, and in DI and 0.1 M ionic strength water. Total bacteria were concentrated from pond water and wastewater samples.

**Fig. 6:**
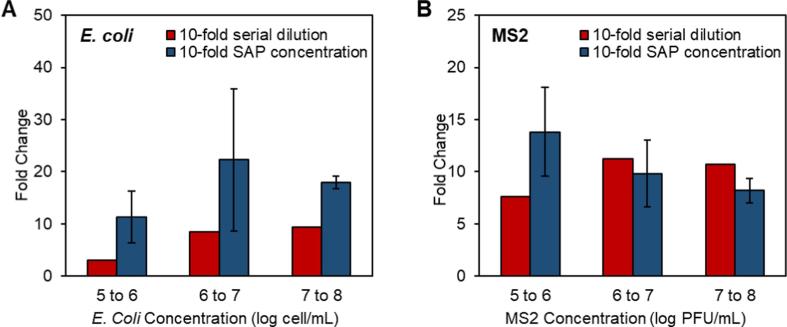
Fold Changes of qPCR and RT-qPCR of *E. coli* (A) and MS2 (B) for samples in varying magnitude of orders with serially diluted samples (red bars) and concentrated samples (blue bars) using the tube concentration system; wherein standard deviations (error bars) were calculated from fold change values of triple independent concentration experiments. Fold change values were calculated from quantification data according to the standard curve performed on each plate. (For interpretation of the references to colour in this figure legend, the reader is referred to the web version of this article.)

The concentration factor (hereinafter referred to as the ratio of the sample volumes before and after the concentration) of SAP microspheres were maintained in a range of 1.3–2.1 for each step, so that the swollen SAP microspheres could be suspended after the concentrating step. When the concentration factor exceeded 4, the concentration efficiency decreased substantially due to that the microorganisms trapped in remaining liquids on the microsphere surface and/or in the voids among the microspheres. The concentration efficiency dropped to 38% when the concentration factor increased an order of magnitude [27]. When using the hand-pressed centrifuge centrifuging step, the concentrate was transferred to the collection chamber. This step substantially improved the concentration factors (the ratio of the sample volumes before and after concentration) and concentration efficiencies. A concentration efficiency of 87 ± 6% was achieved with a concentration factor of 9–10 for *E. coli* in DI water within 20 min (see Fig. 5). By using different SAP formulations, we were able to achieve similar concentration efficiencies of *E. coli* in water with high ionic strengths up to 100 mmol⋅L^−1^. S2 SAP microspheres were used for the concentration of *E. coli* in 100 mmol⋅L^−1^ ionic strength water and an average of 89 ± 17% concentration efficiency was achieved. Additionally, qPCR targeting 16S rRNA gene and RT-qPCR were respectively performed to evaluate the concentration efficiencies of *E. coli* and MS2. As shown in Fig. 6, the fold change values between 10-fold concentrated samples and original samples were found to be 11.34, 22.27 and 17.97, respectively, from *E. coli* solutions with initial concentrations of 10^4^, 10^5^ and 10^6^ cell·mL^−1^. As positive controls, the fold changes between *E. coli* solutions of 10^5^ and 10^4^, 10^6^ and 10^5^, 10^7^ and 10^6^ cell·mL^−1^ were 3.03, 8.50 and 9.34, respectively, which implied the concentration efficiencies of SAP microsphere-based concentration system were respectively 275%, 162% and 92% higher than they were supposed to be by qPCR assays. For the samples of 10^4^, 10^5^ and 10^6^ cell·mL^−1^. Fold change values between samples of 10^5^ (both concentrated and serially diluted) and 10^4^ cell·mL^−1^ were relatively low because the concentration of 10^4^ cell·mL^−1^ is much close to the detection limit of 16S rRNA qPCR. Our results showed that the tube concentration system based on SAP microspheres could achieve satisfactory concentration efficiencies of *E. coli* solutions with a range of initial concentrations.

The bacterial concentrations of original samples did not affect the concentration efficiency as evaluated by microscopic cell counts. Experimental results showed very similar concentration efficiencies (between 85% and 90%) for water samples with different initial concentrations from 10^4^ to 10^8^ cells⋅mL^−1^, thus allowing total concentration efficiencies of higher than 60% for 100- or 1000-time concentration, although 2 or 3 sequential concentration steps may be required. It should be noted that these sequential concentration steps may require multiple formulations of SAP microspheres due to the increasing ionic strength during concentration. It’s extremely difficult to achieve 100–1000 times concentration in one step due to the difficulty in concentrated sample collection and the sample loss on the microspheres’ surface.

Concentration tests using bacteriophage MS2 resulted in a similar level of concentration efficiency (see Fig. 5) evaluated by plaque forming unit quantification. The average concentration efficiency of one concentration step was 101 ± 12% in DI water using O1 SAP. For a 100-mmol⋅L^−1^ ionic strength water sample, the concentration efficiency of MS2 was 90 ± 10%, using S2 SAP microspheres (Fig. 5). The value of >100% was likely caused by the well-known large standard deviation of the double agar layer method, imprecisions in experimental procedures and the MS2 aggregation during experiments. RT-qPCR was performed to evaluate the recovery rates of MS2. As shown in Fig. 6, the fold changes between concentrated samples and original samples were found to be 13.81, 9.83 and 8.20, respectively, for the samples with initial concentrations of 10^4^, 10^5^ and 10^6^ PFU·mL^−1^. Meanwhile, the fold change values between 10^6^ and 10^5^, 10^7^ and 10^6^, 10^8^ and 10^7^ were 7.64, 11.22 and 10.69, respectively, which implied the concentration efficiencies of SAP microsphere-based concentration system were respectively 180%, 88% and 77% comparing to what they were supposed to be by qPCR assays. Fold change values between 10-fold concentrated MS2 samples and original samples are similar to fold change values of between positive control MS2 samples with 10-fold dilution, indicating high concentration efficiencies of the tube concentration system. In summary, results from qPCR and RT-qPCR assays indicate that the SAP microsphere-based concentration method completely meets the requirements for nucleic acid amplification-based environmental monitoring and surveillance. It should be noted that compared to conventional virus concentration methods, such as ultracentrifugation, electropositive or electronegative filters or ultrafiltration [42], [43], [44], the SAP microspheres concentration method neither uses complicated instruments or expensive filters, nor requires the preconditioning of water samples.

Furthermore, the concentration efficiencies of SAP microspheres used for concentrating the native bacteria in the Caltech pond water (ionic strength 15 mmol⋅L^−1^, pH = 7.75) and the wastewater from the wastewater treatment plant (ionic strength 20 mmol⋅L^−1^, pH = 8.02) were investigated. As shown in Fig. 5, average bacterial concentration efficiencies of 112% and 83%, respectively, were achieved for pond water and wastewater samples. The concentration processes were completed in less than 20 min. Presence of other substances in real water samples such as natural organic matters or algae would not influence the performance of our system according to our tests on real environmental waters, which was discussed in Section 3.4.

It should be noted that we introduced itaconic acid to our customized SAP formula to add a negative surface charge and minimize the electrostatic adsorption of microorganisms. Although bacteria and viruses may not always have negative surface charge in environmental waters, which depends on their isoelectric points [45], [46]. As most bacteria have low isoelectric points and will be negatively charged in environmental waters [45], [47], they should be repelled by the SAP beads as what happened to our model bacterium *E. coli*. However, viruses have a broader range of isoelectric points [46]. Our model virus, MS2, has a low isoelectric point (~3.5) [46] and thus, a high concentration efficiency is expected due to electrostatic repulsion. Although accounting for a small part, there are still viruses whose surface charges in natural water may not be strong enough for electrostatic repulsion and therefore the concentration efficiency might be impaired, e.g., somatic coliphage ΦX174 (isoelectric point ~ 7) [46].

### 3.5 Reusability of SAP microspheres

Reusing the microspheres can significantly decrease the cost of our concentration system. After use, the microspheres can be washed and dried for subsequent applications requiring sample concentration. Simple washing with running tap water was sufficient for the reuse of SAP microspheres, as no bacteria or viruses were detected using membrane filtration from the final washing water before the next use. For more sensitive applications, SAP microspheres could be autoclaved as well. To demonstrate their reusability, the SAP microspheres were dried and rehydrated for more than 20 times. Fig. S3 shows the weight change of 100 SAP microspheres for 20 cycles of full drying and swelling. For 20 cycles, the weight change for both dried and swollen microspheres was less than 5%, whereas the decrease of water absorbency was less than 2%. The concentration efficiencies of *E. coli* and MS2 using recycled microspheres (after 20 cycles) were still up to 84 ± 7% and 90 ± 11%, respectively (Fig. 5). Slight efficiency losses during reusing recycled microspheres were most likely attributed to the inevitable breaks of some SAP microspheres during the recycling process, which became much more severe with the increase of recycling times as observed. Damaged spheres might trap much more pathogens due to the increased surface area.

## 4 Conclusion

In this study, tailored SAP microspheres coupled with a hand-powered tube system were developed to achieve efficient and rapid concentration for environmental microorganisms. In order to overcome the performance loss of SAP in high ionic strength water samples, we have been able to improve the water absorption ability of SAP microspheres by optimizing the degree of polymer cross-linking and controlling the counter ion concentrations using the Flory model as a guide. Optimally synthesized SAP microspheres were shown to absorb more water at higher absorption rates compared to other commercially available water-absorbing microspheres, making our synthetically-tailored SAP microspheres able to concentrate bacteria and viruses from high ionic strength water samples and environmental water samples within a short time. In addition, we developed a low-cost, portable, hand-powered portable centrifuge tube system based on our tailored SAP microspheres to facilitate concentrating water in low-resource settings in the field. Results from our study highlight that we provide a cost-effective, easy-to-use and off-grid system with tailored SAP microspheres for various water samples. We envision that this system could be applied to the field for efficient microbial concentration and promote rapid on-site microbial analysis.

## CRediT authorship contribution statement

**Xunyi Wu:** Conceptualization, Methodology, Investigation, Formal analysis, Writing - original draft. **Xiao Huang:** Conceptualization, Methodology, Writing - review & editing. **Yanzhe Zhu:** Writing - review & editing. **Jing Li:** Methodology, Writing - review & editing. **Michael R. Hoffmann:** Conceptualization.

## Declaration of Competing Interest

The authors declare that they have no known competing financial interests or personal relationships that could have appeared to influence the work reported in this paper.
