# Supplementary material for: Synthesis and application of superabsorbent polymer microspheres for rapid concentration and quantification of microbial pathogens in ambient water
Source: Sep Purif Technol. 2020 May 15;239:116540. doi: 10.1016/j.seppur.2020.116540 (PMC7045201; doi:10.1016/j.seppur.2020.116540)
Supplement: Supplementary data 2 [file mmc2.docx]

**Supporting Information**

**Synthesis and Application of Superabsorbent Polymer Microspheres for Rapid Concentration and Quantification of Microbial Pathogens in Ambient Water**

Xunyi Wu, Xiao Huang, Yanzhe Zhu, Jing Li, and, Michael R. Hoffmann^*^

Linde+ Robinson Laboratories, California Institute of Technology, Pasadena, California 91125, United States.

^*^ Correspondence and requests for materials should be addressed to MRH

Tel: 626-395-4391 EM: mrh@caltech.edu.

**Submitted to: *Separation and Purification Technology***

**December 2019**

Number of pages: 12

Supporting information contains 7 figures and 3 tables

# 1. Fabrication of poly(acrylamide-co-itaconic acid) beads using inverse suspension polymerization in a batch reactor

At room temperature, 40 mL of water was added to a well-mixed oil phase containing 185 mL cyclohexane, 65 mL tetrachloroethylene, 0.75 g Span-80 and 0.375 g Tween-60 in a 500-mL flask. The stirring speed was set to be 180 rpm and the water phase was inverse suspended into the oil phase by stirring. At the same time, the flask was heated by a water bath to 80°C. When the temperature is reached, the system was kept at this temperature for the full polymerization of the water phase. After two hours, heat was removed, and when the system was cooled to below 50°C, SAP beads were precipitated by adding ethanol. Harvested beads were washed with pure ethanol and dried in a vacuum oven overnight.

# 2. Water absorption rate of the SAP beads and model fits

To investigate the influence of monomer composition on the water absorption rate of SAP, three models for diffusion of water into a SAP spheres have been applied to evaluate the absorption performance. Expressions for radius expansion over time by these models are summarized in Table S2. Data of the swelling behavior of i) low sodium SAP bead in DI water, ii) low sodium SAP bead in 0.1M ionic strength water, iii) high sodium SAP beads in 0.1M ionic strength water are plotted and fitted to these three models through least squares fitting (Fig. S2).

In these models, a diffusion coefficient is used as a fitting parameter. According to the assumptions and calculations used by these models, corresponding diffusion coefficients do not have the same units and scales for these three models, but we can compare the best fitting diffusion coefficients within each model to imply the rate of diffusion of different SAP spheres in different water samples. These three models do not provide prefect fits for our experimental data, but the general curves for the radius change over time predicted by the models correlate with our experimental data. By reading the best-fitting diffusion coefficients, most models show a decrease in diffusion coefficients when the SAP swells in water with higher ionic strength, which can be accounted for by the reduced cation concentration difference inside and outside the SAP sphere, and thus the reduced osmotic force. However, when increasing the sodium content in SAP, all models show that the diffusion coefficients increase by around 50%, which is conform to the increase of osmotic force.

The fit of these models to our experimental data is not perfect mainly for two reasons. First, the diffusion of water into SAP spheres involves the decrease of osmotic force, increase of the polymer retention force, and the electrostatic force between negatively-charged polymer chains, which is much more complex than what the models can describe. Also, all models only adopt one parameter which decreases the flexibility of these models. Therefore, to better describe the swelling behavior of SAP, a more detailed model would have to be developed.

**
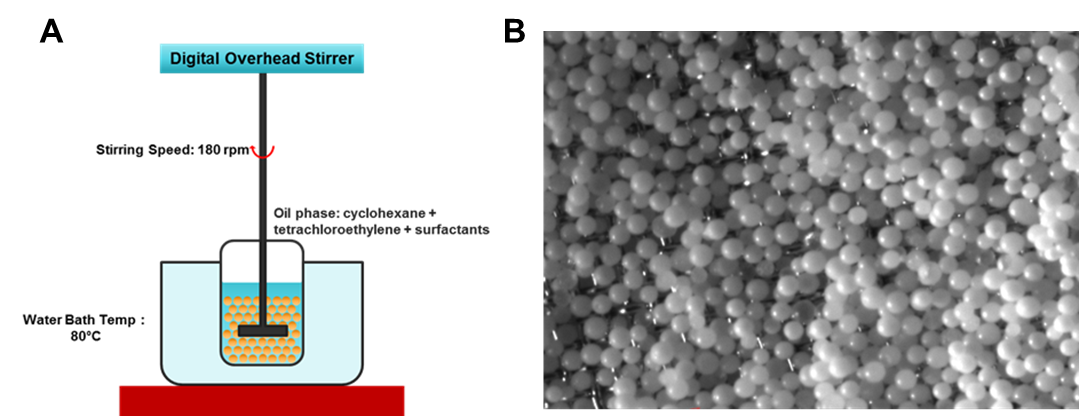
**

**Figure S1.** A) Schematics of the fabrication process of SAP beads using inverse suspension polymerization in a batch reactor. B) microscope image of beads fabricated by inverse suspension polymerization with average diameter of 120 µm.

*
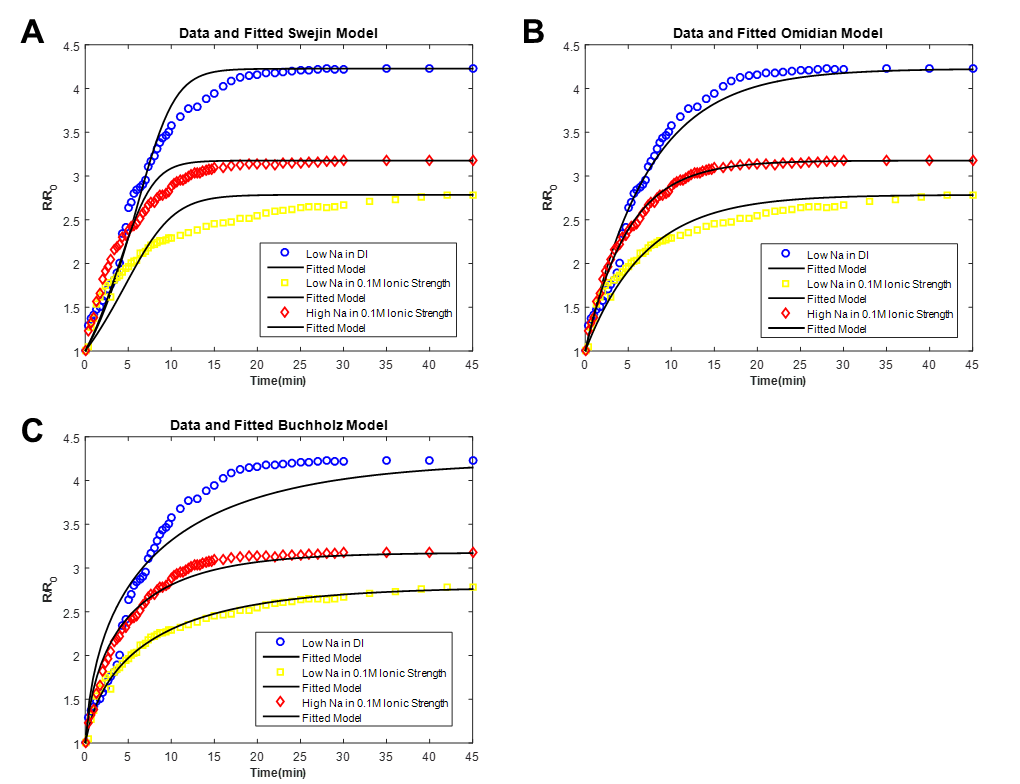
*

**Figure S2.** Comparison of experimental data and the three employed models to evaluate the absorption rate of SAP beads (diameter change verses time).

**
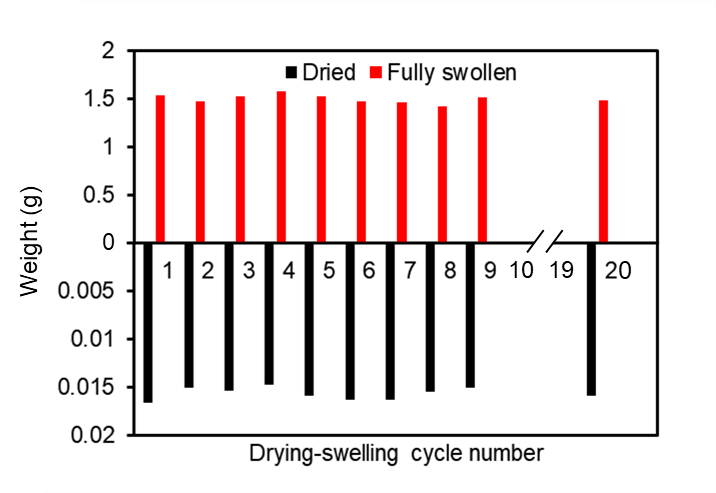
**

**Figure S3.** Weight change of 100 SAP beads for 20 drying-swelling cycles. Their mass when completely dried and swollen was measured and compared and no weight or water absorbency loss was observed.

**
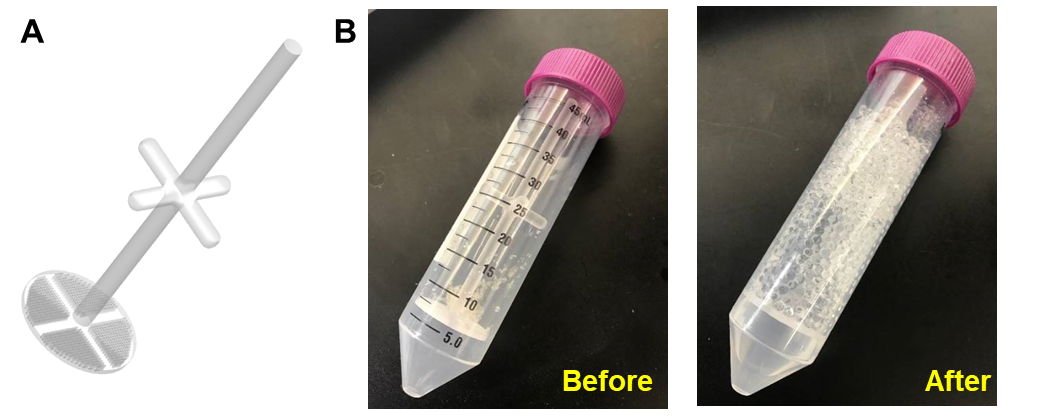
Figure S4.** A) Design of the filter in the tube system. The filter has a mesh size of 300 μm, and was fabricated by 3D-printing. B) Pictures of the tube system before and after use. Dried SAP beads were pre-loaded in the tube. When the concentration was completed, concentrated sample is ready to be collected in the lower chamber.


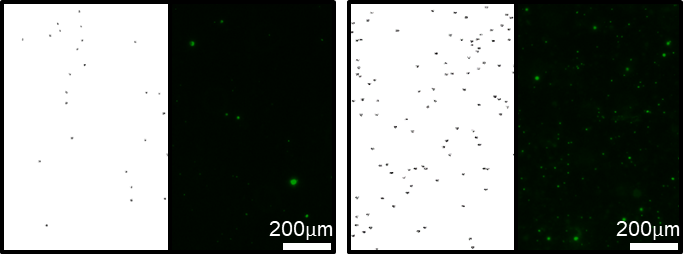


**Figure S5.** Fluorescence microscope images of *E. coli* concentration before and after concentration. The left side of the images were processed by ImageJ for counting. For a 10-fold concentration, an average recovery efficiency of 87% is achieved.

**
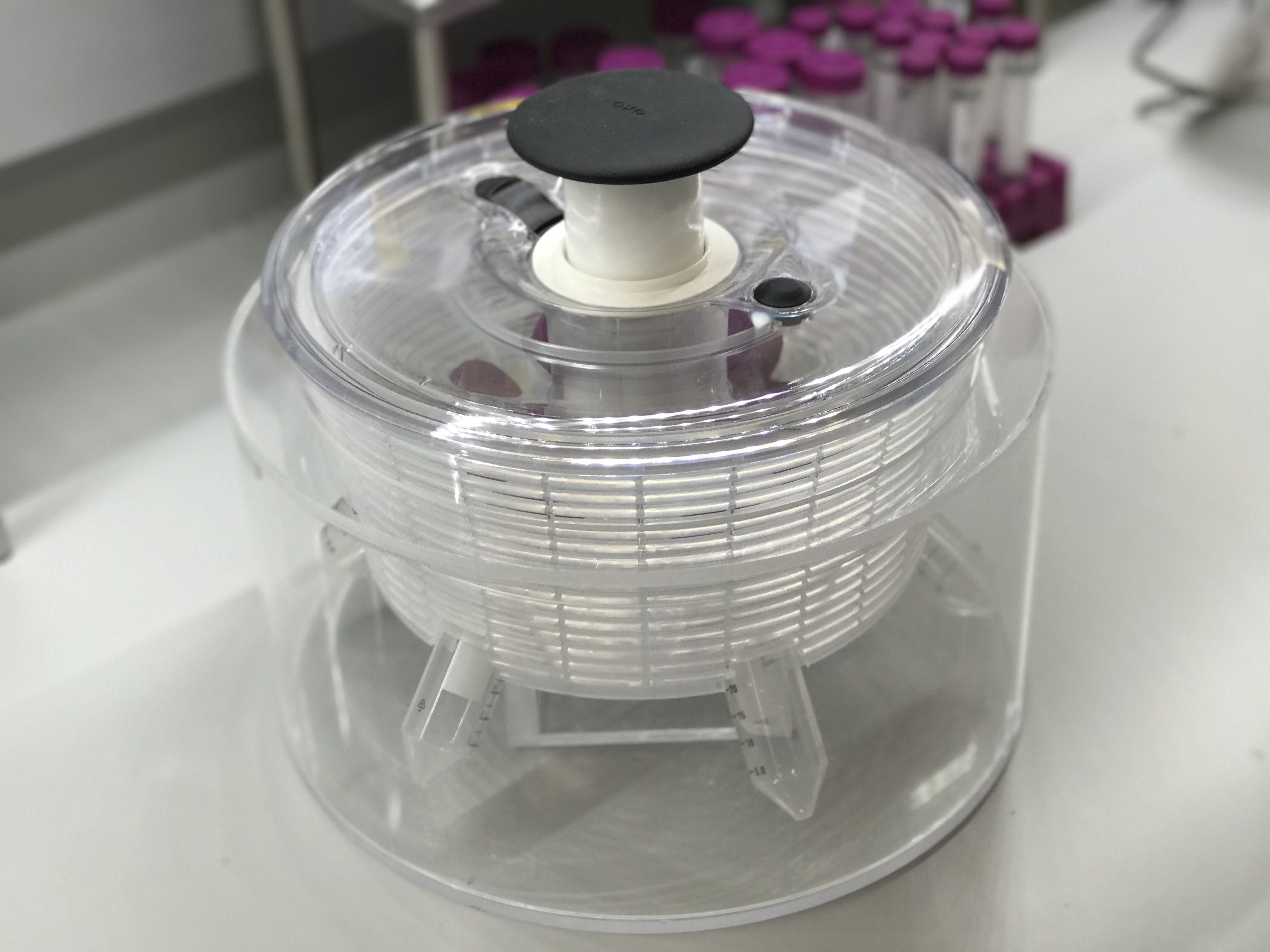
**

**Figure S6.** Centrifuge adapted from a salad spinner for concentration tests.


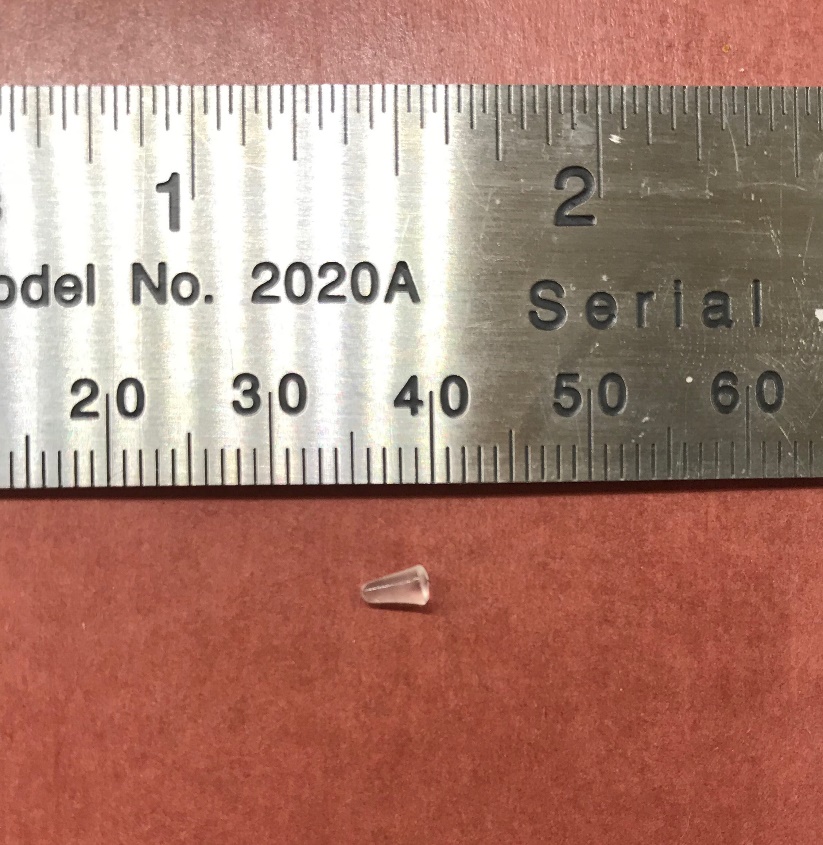


**Figure S7.** Fabricated SAP blocks for water absorption studies.

**Table S1-1. Primer and probe sequences for the 16s rRNA qPCR assay^1^**

| 16s rRNA Primer/probe | Sequence (5’‐3’) |
| --- | --- |
| Forward primer | CGGTGAATACGTTCYCGG, where Y is either C or T |
| Reverse primer | GGWTACCTTGTTACGACTT, where W is either A or T |
| TaqMan probe | FAM-CTTGTACACACCGCCCGTC |

**Table S1-2. MS2 primer and probe sequences for the RT‐qPCR assay^2^**

| MS2 Primer/probe | Sequence (5’‐3’) |
| --- | --- |
| Forward primer | ATTCCGACTGCGAGCTTATT |
| Reverse primer | TTCGACATGGGTAATCCTCA |
| TaqMan probe | FAM‐ ATTCCCTCAGCAATCGCAGCAAACT‐ BHQ1 |

**Table S2.** Models used to evaluate the swelling of one SAP sphere:

| Equation | Reference |
| --- | --- |
| $\frac{\boldsymbol{R}}{\boldsymbol{R}_{\boldsymbol{0}}}\boldsymbol{=}\left( \frac{\boldsymbol{R}_{\boldsymbol{m}}}{\boldsymbol{R}_{\boldsymbol{0}}}\boldsymbol{-1} \right)\left( \boldsymbol{1-}\boldsymbol{e}^{\boldsymbol{-kt}} \right)\boldsymbol{+1}$ | Omidian et al. (1998)^3^ |
| $\frac{\boldsymbol{dQ}}{\boldsymbol{dt}}\boldsymbol{=}\frac{\boldsymbol{\pi}^{\boldsymbol{2}}\boldsymbol{D}}{\boldsymbol{R}_{\boldsymbol{0}}}\boldsymbol{(}\boldsymbol{Q}_{\boldsymbol{max}}\boldsymbol{-}\boldsymbol{Q}_{\boldsymbol{o}}\boldsymbol{)}$ | Buchholz (1998)^4^ |
| $\frac{\boldsymbol{R}}{\boldsymbol{R}\boldsymbol{0}}\boldsymbol{=}\sqrt[\boldsymbol{3}]{\frac{\boldsymbol{1}}{\boldsymbol{1-\theta}\left( \boldsymbol{t} \right)}}$  $\boldsymbol{where \theta}\left( \boldsymbol{t} \right)\boldsymbol{=}\boldsymbol{\theta}_{\boldsymbol{max}}\boldsymbol{-}\left( \boldsymbol{\theta}_{\boldsymbol{max}}\boldsymbol{-}\boldsymbol{\theta}_{\boldsymbol{0}} \right)\boldsymbol{e-kt}\boldsymbol{ereee}\boldsymbol{e to evaluate the swelling of SAP sphere:}$ | Sweijen et al. (2017)^5,6^ |

*Note: Q in Buchholz’s model represents the mass of absorbed water to the mass of dry SAP, which can be rewritten in terms of R (assuming water is incompressible), and R(t) can be yielded by numerical integration.*

*E. coli*

| Before concentration (cell/mL) | After concentration (cell/mL) | | | | |
| --- | --- | --- | --- | --- | --- |
|  | #1 | #2 | #3 | Mean | STD |
| 3.95E+04 | 2.33E+05 | 4.93E+05 | 6.21E+05 | 4.49E+05 | 1.98E+05 |
| 1.20E+05 | 1.46E+06 | 2.01E+06 | 4.52E+06 | 2.66E+06 | 1.63E+06 |
| 1.02E+06 | 1.29E+07 | 1.74E+07 | 2.45E+07 | 1.83E+07 | 5.85E+06 |

MS2

| Before concentration (PFU/mL) | After concentration (PFU/mL) | | | | |
| --- | --- | --- | --- | --- | --- |
|  | #1 | #2 | #3 | Mean | STD |
| 1.15E+05 | 2.07E+06 | 1.09E+06 | 1.61E+06 | 1.59E+06 | 4.90E+05 |
| 8.80E+05 | 1.19E+07 | 6.88E+06 | 7.19E+06 | 8.66E+06 | 2.81E+06 |
| 9.88E+06 | 8.85E+07 | 7.14E+07 | 8.30E+07 | 8.10E+07 | 8.73E+06 |

**Table S3.** Quantification data of samples before and after concentration experiments using qPCR and RT qPCR for *E. coli* and MS2. All concentration experiments were performed as individual triplicates, and all samples were run as triplicates on the plate. Standard curves with known gradient concentrations were run on each plate for the quantification with reaction efficiency between 90% and 110%

# References:

(1) Suzuki, M. T.; Taylor, L. T.; DeLong, E. F. Quantitative Analysis of Small-Subunit RRNA Genes in Mixed Microbial Populations via 5’-Nuclease Assays. Appl. Environ. Microbiol. 2000.

(2) Huang, X.; Lin, X.; Urmann, K.; Li, L.; Xie, X.; Jiang, S.; Hoffmann, M. R. Smartphone-Based in-Gel Loop-Mediated Isothermal Amplification (GLAMP) System Enables Rapid Coliphage MS2 Quantification in Environmental Waters. Environ. Sci. Technol. 2018, 52 (11), 6399–6407.

(3) Omidian, H.; Hashemi, S. A.; Sammes, P. G.; Meldrum, I. Modified Acrylic-Based Superabsorbent Polymers (Dependence on Particle Size and Salinity). Polymer (Guildf). 1999, 40 (7), 1753–1761.

(4) Buchholz, F. L.; Graham, A. T. Modern Superabsorbent Polymer Technology; 1998.

(5) Sweijen, T.; van Duijn, C. J.; Hassanizadeh, S. M. A Model for Diffusion of Water into a Swelling Particle with a Free Boundary: Application to a Super Absorbent Polymer Particle. Chem. Eng. Sci. 2017, 172, 407–413.
